# Weight Loss Strategies and the Risk of Skeletal Muscle Mass Loss

**DOI:** 10.3390/nu13072473

**Published:** 2021-07-20

**Authors:** David McCarthy, Aloys Berg

**Affiliations:** 1 Public Health Nutrition Research Group, London Metropolitan University, London N7 8DB, UK; 2 Faculty of Medicine, University of Freiburg, 79117 Freiburg, Germany; aloys.berg@klinikum.uni-freiburg

**Keywords:** obesity, weight loss, interventions, body composition, fat-free mass, skeletal muscle mass, exercise, sarcopenia, protein intake

## Abstract

With energy intake restriction and exercise remaining the key diet and lifestyle approaches to weight loss, this is not without potential negative implications for body composition, metabolic health, and quality and quantity of life. Ideally, weight loss should be derived almost exclusively from the fat mass compartment as this is the main driver of metabolic disease, however, several studies have shown that there is an accompanying loss of tissue from the fat-free compartment, especially skeletal muscle. Population groups including post-menopausal women, the elderly, those with metabolic disease and athletes may be particularly at risk of skeletal muscle loss when following a weight management programme. Research studies that have addressed this issue across a range of population groups are reviewed with a focus upon the contribution of resistance and endurance forms of exercise and a higher intake dietary protein above the current guideline of 0.8 g/kg body weight/day. While findings can be contradictory, overall, the consensus appears that fat-free and skeletal muscle masses can be preserved, albeit to varying degrees by including both forms of exercise (but especially resistance forms) in the weight management intervention. Equally, higher intakes of protein can protect loss of these body compartments, acting either separately or synergistically with exercise. Elderly individuals in particular may benefit most from this approach. Thus, the evidence supports the recommendations for intakes of protein above the current guidelines of 0.8 g/kg body weight/d for the healthy elderly population to also be incorporated into the dietary prescription for weight management in this age group.

## 1. Introduction

Restriction of energy intake to below energy requirements coupled with increased levels of physical activity (both aerobic and resistance/strength forms) remain the key non-surgical and non-pharmacological therapeutic weight loss strategy. This is important for achieving clinically meaningful weight loss for health as well as for performance purposes in athletes and active people. The health benefits of weight loss include improvements in insulin sensitivity and glycaemia, lower blood pressure, and better blood lipid profile [1]. Whilst the desired weight loss should arise from an almost exclusive loss of fat mass (FM, since this tissue is a key driver of metabolic risk), it is inevitable than some unintentional loss of mass will also occur from the lean or fat-free mass (FFM) compartment including skeletal muscle mass (SMM). In general, typical losses of total body weight comprise between 20–40% FFM, the remainder coming from FM. This apparent indiscriminate loss of body tissue can have potential negative effects for both short- and long-term health and physical function, but also for performance in athletes and active people. Thus, optimum weight loss strategies should aim to preserve, as best as possible, the whole-body SMM. This review will briefly address the metabolic basis for the loss of skeletal muscle (SM) during weight loss followed by an examination of the literature on the dietary and physical activity strategies to minimise or reduce the loss of FFM/SMM.

## 2. Skeletal Muscle-Function, Metabolism, Health, and Mortality

SM mass and strength are key to the architectural structure of the human body by supporting the frame. It also generates contractile forces for locomotion/ambulation and other movements that are essential for independent living [2]. Furthermore, SM is increasingly being recognised as an independent marker of metabolic health [3], where low muscle fitness is associated with metabolic risk, muscle mass, and strength being inversely related to later CVD risk and mortality and muscular strength being positively related to higher insulin sensitivity. SM is normally responsible for more than 75% of all insulin-mediated glucose disposal and given the relative size of the whole-body SMM, is thus a key tissue in whole-body glucose homeostasis. Insulin resistance at this site is a particularly important driver of risk for type 2 diabetes [4]. SM is also a major site of lipid oxidation where it has a unique role in modulating whole-body fat balance via aerobic metabolism [5]. Furthermore, given its mass and metabolic activity, SM contributes to a substantial fraction of resting energy expenditure and hence energy demands [6]. An increasing number of studies are reporting an association between SMM and strength with morbidity, longevity, and mortality [7,8,9]. In 2002, Metter et al. used hand grip as a measure of muscle strength and survival analysis over a 40-year period [10]. They found that lower and declining grip strength was associated with increased mortality. A 2018 study exploring the relationships between body composition and all-cause mortality demonstrated that participants with low muscle mass not only had higher body fat percentage, but more importantly, an increased likelihood of diabetes and higher adjusted mortality [11]. In a population of Chinese elders, the association between SMM and long-term all-cause mortality was evaluated with the findings indicating that low muscle mass was a predictor of long-term mortality in nonagenarian and centenarian women, but not in men [12]. A 2019 Brazilian study investigated the association between body composition assessed by dual-energy X-ray absorptiometry, (DXA) and mortality in a cohort of elderly subjects. The presence of low muscle mass significantly increased all-cause mortality risk in men and similar results were observed for cardiovascular mortality. In women, low muscle mass was a predictor of all-cause and cardiovascular death [13]. Similar findings have been observed in the U.S. in older adults [9], whereas an earlier study had shown that muscle strength but not muscle mass was associated with mortality [14]. However, it should also be noted that not all studies have been able to demonstrate an association between SM and mortality after adjustment for potential confounders [15]. Furthermore, a recent study compared the prognostic value of body mass index (BMI) versus appendicular SMM (aSMM) and FM with fatal and non-fatal cardiovascular disease and all-cause mortality [16]. Their study showed that while FM showed a strong positive association with CVD risk, for aSMM, this risk differed between the sexes and that measurements of FM and aSMM were not superior to BMI in predicting CVD incidence or all-cause mortality at the population level. Taken together, these observations demonstrate that any loss of SM could impair quality (and quantity) of life, and maintaining an optimum SMM is important for long-term health, physical function, and longevity.

## 3. Weight Gain, Obesity, and Skeletal Muscle Mass

When body weight is gained during a state of positive energy balance, this is predominantly due to gains in FM but there is also some expansion of the muscle mass [17,18]. This gain has been considered necessary, in part, to structurally support the extra weight of the FM both statically and during ambulation. A compensatory gain in muscle mass to counteract the negative effect of fat on insulin sensitivity is a further consideration. However, as a consequence of the ageing process, chronic inactivity or the result of repeated attempts at weight loss, a condition known as sarcopenic obesity can arise [19]. In this situation, even though a healthy BMI may be apparent, this masks an imbalance between (excess) body fat and (decreased) SM. This has a number of consequences including a reduction in resting metabolic rate, a reduced capacity for physical activity, and increased risk for metabolic disease.

## 4. Regulation of SMM Maintenance and Turnover

SMM varies across the life cycle with gains during the growth phases across childhood and adolescence [20]. In adulthood, weight maintenance, weight gain, and weight loss all impact upon SMM and as a result of the ageing process, SMM tends to decrease, where in some individuals, this rate of loss can tip them into a critical state of sarcopenia [21]. At the cellular level, SMM status reflects the interrelationship between myofibrillar and cytoplasmic protein synthesis (muscle protein synthesis (MPS) and degradation (muscle protein breakdown MPB), known as skeletal muscle protein turnover. Reflecting both the nutritional environment and physical activity stimulus (either resistance or aerobic), a collection of anabolic hormones act in concert to promote synthesis (insulin, testosterone, growth hormone, IGF-1) and breakdown or catabolism (cortisol, glucagon, adrenaline). Experimental molecular markers of SM synthesis include stable isotopes incorporated into specific amino acids (e.g., ^15^N-glycine or ^15^N-leucine) and relates to the nutritional regulation of SMM turnover [22]. Beyond the endocrine regulation of SMM as outlined above, a range of factors are known to regulate SMM. Whilst undernutrition (both energy and protein) clearly impacts upon SMM (see below) as well as ageing (sarcopenia, dynapenia) and pathology (cachexia), both nutrition (feeding) and exercise also regulate SMM via their effects upon SM protein turnover [2,22]. In short, under basal, post-absorptive (fasting) conditions, MPB exceeds MPS whereas MPS is stimulated post-prandially. Thus, following consumption of a meal, the dietary-derived amino acids can compensate for any losses of SM amino acids by driving up MPS as well as acting synergistically with insulin to suppress MPB. At the molecular level, protein deposition in skeletal muscle and hence tissue hypertrophy (through an increase in muscle protein synthesis) is driven by amino acid, insulin, and IGF-1-mediated increases in ribosomal capacity via the rapamycin complex (mTOR) signalling pathway. mTOR is a kinase, encoded by the mTOR gene in humans. Intense exercise also activates mTOR signalling and can amplify the effect of dietary-derived amino acids on this pathway [20].

Furthermore, the amount of protein consumed determines the SM protein gain via a dose-dependent essential amino acid stimulation of MPS. Beyond a certain threshold of dietary protein consumption (~0.24–0.4 g/kg body weight in a single meal), the rate of amino acid oxidation increases without any further increase in MPS [23]. Critically, resistance exercise also influences SM protein turnover, with weight training promoting SMM increase largely due to an increase in MPS as opposed to suppression of MPB. Furthermore, evidence suggests that the effects of resistance exercise and protein ingestion on MPS are additive [24]. In this context, protein consumption following resistance exercise enhances MPS (and hypertrophy). Here, the provision of EAAs is key, with leucine supply of particular importance. Indeed, hyperleucinaemia is required to promote MPS, with leucine-rich protein foods appearing to be the most effective, post resistance exercise [25].

Historically, biochemical markers of SM protein breakdown include the measurement of the excretion of the metabolite 3Me-Histidine [26]. 3Me-His is a unique component of the skeletal muscle contractile protein actin. Upon actin degradation, 3-Me-His is excreted unchanged in urine and is not reincorporated back into new body proteins. Thus, a rise in 3Me-His excretion is an indication of SMM breakdown and is observed in catabolic states [27]. Similarly, 24 h creatinine excretion has long been used as a biochemical marker of SMM. Creatinine is the degradation product of creatine—a short-term energy storage compound present almost uniquely in SM. Approximately 98% of whole-body creatine is found in skeletal muscle and is excreted at a constant daily rate (once any dietary creatine has been accounted for) in proportion to the whole body SMM [28]. 

The breakdown of SM resulting from therapeutic weight loss and a state of negative energy balance is reflected in a short-term negative nitrogen balance with an increase in the excretion of nitrogen, predominantly as urea, the end product of amino acid degradation [29]. Whilst it is preferable for long-term metabolic and physical health that SMM is maintained (as well as for performance purposes during sport and exercise), it needs to be recognised that SM loss can result from a demand for the mobilisation of amino acids as a precursor for gluconeogenesis in an energy deficient state [30]. Equally, when total body mass falls as a result of body fat loss, there should be a resultant lower demand for the structural and architectural support for a heavier body mass, hence a likely lower need for the raised SMM in obesity.

Current approaches to the quantitative assessment of SMM include segmental bioimpedance systems and scanning technologies including DXA, magnetic resonance imaging, (MRI), and computerized tomography (CT) [31]. However, given the potentially slow rate of SMM loss across the period of therapeutic weight loss, both bioelectrical impedance analysis (BIA) and DXA could fail to detect small losses over a short time period. Air-displacement plethysmography quantifies FM and FFM via assessment of body density. While it is unable to partition FFM into SM and non-SM masses, nevertheless, this technology can be used to track losses of FFM (where it is anticipated that the loss will come predominantly from the SMM) across a phase of weight management intervention. The following aspect of this review will evaluate the scale of SMM loss during therapeutic weight loss by using these markers and measures of SMM.

## 5. Fat-Free Mass and Skeletal Muscle Mass Loss Following Therapeutic Dietary Weight Loss Interventions

With the increased access to improved body composition assessment technology, the extent and variation in FFM and SMM loss following weight management intervention can be better quantified. However, several factors could influence FFM and SMM loss including the initial degree of excess body weight (BMI), duration and intensity of intervention, dietary approach, involvement of physical activity and exercise components, age of participant as well as sex and ethnicity differences. This range of influencing variables can therefore make it difficult to generalise on the extent of SM that is lost over the course of a weight management intervention. To further compound this issue, SM and changes in SM can be expressed in a variety of measures that include muscle volume, muscle cross-sectional area, skeletal muscle index (SMI, SMM kg/height m^2^) as well as the simpler whole-body SM (g or kg) or appendicular SMM (aSMM) [32]. Nevertheless, several studies have attempted to quantify both FFM and SMM loss following dietary intervention. Unsurprisingly, results are highly variable and are dependent upon several factors including the duration of the intervention, intensity of the intervention, dietary (and exercise) approach, population group, and body composition assessment technique. Reported losses range between 1.0 and 1.5 kg FFM and in the region of around 3.2% SMM or 0.9 kg–1.7 kg [33,34,35,36,37]. A number of studies have evaluated the effectiveness of a low-carbohydrate approach to weight loss with a special focus upon the preservation of FFM/SMM [38,39,40]; Meckling et al. (2004) found that preservation of FFM was better after a 10-week intervention when following a low-fat/low-energy diet compared with a low-carbohydrate/low-energy diet [38]. Volek et al. (2004) compared a very-low carbohydrate/ketogenic diet against a more conventional low-fat diet in a group of 28 volunteers aged 20–55 y. FFM preservation was better in the males following the low-fat diet, whereas in females, the reverse was observed. However, it should be noted that the duration of the intervention was shorter (30 days) in the females compared to the males (50 days) [39]. In contrast, Volek et al. (2010) showed that when consuming a diet restricted in carbohydrate but with a higher protein level, coupled with resistance exercise, a better preservation of the FFM was observed. Here, the inclusion of the training element appears to be key to FFM maintenance [40]. In one study that used a meal replacement product as part of the weight management intervention and where body composition changes were assessed using air-displacement plethysmography, they found evidence of a lean tissue preservation ascribed to the high soy protein, low fat nature of the meal replacement product [41]. Sex differences in both the degree and composition of weight loss in an overweight and obese population on similar weight loss regimens have been observed, with greater changes in FFM than in FM for men than for women (assessed by DXA) as well as greater overall weight loss for men than for women [17]. Although the influence of ethnicity on the composition of weight loss has been largely unexplored, there is evidence to suggest that in premenopausal women, compared with African-Americans, those from European-American women, without accompanying resistance exercise, have less initial skeletal muscle and lose more muscle with weight loss [42]. It has been suggested that the hormonal environment may, in part, explain these differences [43].

Caution must be exercised when interpreting these findings due to the difference in the accuracy of the instrumentation used to measure the body compartment and the variation in the study design, sample size, and the profile of the population group. However, it is evident that not all of the FMM lost during diet restriction-based weight management is SM, and thus likely includes loss from connective tissue and viscera, especially liver [36]. It is interesting to note from a 2019 study that when comparing the degree of dietary energy restriction (severe versus moderate over a 12-month period), the severe energy restriction—despite leading to greater overall weight loss and lean tissue loss—had no greater negative impact on relative whole-body lean mass or strength compared with moderate energy restriction [44]. Taken together, the findings from these studies clearly illustrate that FFM and SMM losses do occur as a result of a dietary energy-restricted weight loss regime. 

Mechanistically, from a protein turnover perspective, it remains unclear whether the loss of SM tissue during diet-restricted intervention results from a suppression of protein synthesis or an increase in protein breakdown. A critical aspect could be whether an exercise or physical activity component forms part of the intervention. This element could be aerobic in nature (such as brisk walking or jogging) or strength (resistance) related such as lifting weights. As evidenced below, numerous studies have demonstrated a more favourable loss of FM with the inclusion of aerobic forms of exercise, but a protective effect of resistance exercise on the preservation of FFM and SMM. At the molecular level, it is likely that this protective effect results from a stimulation of MPS, although this is a likely consequence of an augmentation of MPS following protein consumption (see later). However, the combination of a dietary approach with an exercise component should be recognised as a potential intervention to minimise or prevent SMM loss. Discussion of studies relating to this approach now follows.

## 6. Fat-Free Mass and Skeletal Muscle Changes Following Weight Loss with an Exercise Component

Outside the weight management domain, exercise has long been known to impact upon skeletal muscle mass, strength, and composition [2]. Exercise can be simply divided into two main types: resistance or strength forms and aerobic/endurance exercise. Resistance exercise, being predominantly anaerobic in nature, is the behavioural and physiological approach to stimulating SMM growth and expansion in a weight gaining regime (e.g., in body building). Additionally, resistance forms of exercise have also been evaluated in weight management interventions as an additional approach to preserving FFM/SMM. In contrast, aerobic exercise can promote fat loss as part of a weight management intervention [33]. A 2011 study evaluated the effects of weight loss and exercise (aerobic, resistance, flexibility, and balance), independently and combined with body composition (using DXA and MRI), in a group of older (65 y+) obese participants over a period of 12 months [45]. With a substantial weight loss in the intervention groups, lean body mass (LBM) decreased less in the diet + exercise group compared with the diet only group. LBM increased by 1.3 kg in the exercise only group. They concluded that adding an exercise element to a dietary weight loss regime may be the best approach for improving physical function and ameliorate frailty in obese older adults. Foster-Shubert and colleagues (2012) compared the effect of diet and exercise, alone or combined, on weight and body composition (assessed by DXA) in overweight and obese postmenopausal women across a 12-month intervention period [46]. With respect to the lean mass component, this increased among those participating in exercise alone (+0.3 kg). This increase, however, did not differ significantly from women in the control group but it was significantly greater compared to the reductions in lean mass among women in the diet alone (−0.8 kg, *p* < 0.0001) or diet + exercise (−0.4 kg, *p* = 0.003) groups. The authors concluded that the findings could have important implications for older people as they are at an increased risk for sarcopenia, although they did not specifically quantify SMM in the study. With appropriate duration and intensity, it is possible to protect against loss of SMM during weight loss [37]. One population group that may be particularly vulnerable to increased rate of SMM loss whilst undergoing weight management intervention are the elderly. Indeed, without judicious attention to the composition of the weight management programme, older individuals with excess weight may be at risk of developing sarcopenic obesity as a result of unintended excess loss of SMM following a weight management intervention [19,47]. In this context, several weight management and body composition studies have focused specifically on this age group. Frimel et al. (2008) evaluated weight-loss-induced reduction in muscle mass in frail obese older adults [48]. Participants were randomly assigned to six months of diet/behavioural therapy or diet or behavioural therapy plus progressive resistance training. They found that by adding this exercise component to the programme, muscle mass loss was reduced (assessed by DXA), and muscle strength was increased compared with the diet/behavioural approach only. A similar attenuation of muscle mass loss was observed in a group of obese postmenopausal women when a low intensity, resistance exercise training element was added to a hypocaloric diet for a period of 12 weeks [49]. Turning to aerobic exercise, again, a beneficial attenuation of muscle mass loss has been observed in this age group when aerobic exercise, in the form of moderate-intensity walking, 3–5 times per week for 35–45 min was added to a low-fat, 500–1000 kcal/d energy restriction, over a period of four months [50]. In this study, body composition was assessed by DXA, thigh CT, and percutaneous muscle biopsy to quantify fibre type. This latter procedure demonstrated that type 1 (aerobic) fibres decreased in the diet/behavioural group but remained unchanged in the group with exercise included. The same pattern was observed for type 2 (anaerobic) fibres. Nevertheless, the impact of including an exercise element in a weight management programme in an older population does not always achieve the same outcome. Santanasto and colleagues (2010) evaluated the addition of a successful healthy ageing education element to a physical activity (combined strength, aerobic, balance, and flexibility exercises plus weight loss programme. Findings showed that without the education element, loss of muscle area (assessed by CT and DXA) and strength was significantly greater after six months of intervention [51]. A study focusing on Japanese adults aged 40–75 y evaluated the effects of energy restriction with or without aerobic exercise (20 min each of step exercises, bicycle ergometry, and walking or running, 60 min per session, three times per week for 12 weeks) on visceral adiposity and thigh muscle mass. Body composition was assessed by hydrodensitometry and CT scanning [52]. The findings from this study indicated that aerobic exercise attenuated the loss of skeletal muscle during energy restriction in adults with visceral adiposity. It is not only when diet is combined with an exercise element where a preservation of SMM and strength is observed following weight management intervention. In a study of healthy 50–60 year olds (mean BMI 23.5), exercise only-(frequent vigorous endurance exercise) induced weight loss (for 12 months) resulted in better outcomes for lean mass (DXA) muscle volume (MRI) and strength (dynamometry) compared with an energy restricted diet at comparable decreases in body weight [53]. A conclusion from this study suggested that during exercise-induced weight loss, the body is able to adapt to maintain or enhance physical performance capacity. These findings have been replicated and extended to suggest that in this context, exercise is likely to improve physical function [37]. Finally, when comparing exercise interventions performed at low (control), moderate, and intense, Hernández-Reyes et al. demonstrated that in a group of premenopausal women following a hypocaloric diet (−500 kcal/d), after three months, a significant reduction in body weight, BMI, and body fat was observed [54]. Additionally, the intense exercise group demonstrated a significantly much lower reduction in SMM (*p* < 0.05) compared with the other groups. After six months, those in the moderate group showed a less pronounced loss in SMM compared with the low intensity group, whereas those in the intense group showed a small but significant gain in muscle mass (*p* < 0.001). These findings reinforce the importance of an optimum composition of a weight management intervention in older individuals in order to preferentially promote loss of body fat with minimal impact upon SMM. Indeed, the continuation of a higher level of physical activity beyond the weight management phase (most likely a combination of resistance- and aerobic forms of exercise) is likely to support the preservation of SMM and a healthy body composition, and that lower levels of PA may be insufficient to maintain or stimulate any anabolic drive to the tissue. Taken together, the findings from these and other studies have confirmed that loss of SMM is not necessarily an inevitable consequence of a weight management programme and that the inclusion of an exercise element, likely a resistance type or a combination of resistance and aerobic, will contribute to some preservation of lean tissue, especially SM. One further critical factor that needs to be evaluated in detail is the level and quality of dietary protein provided in the weight management regimen. 

## 7. Influence of the Level and Quality of Protein in the Dietary Prescription for Weight Management

In the determination of human protein requirements from both a quantitative and qualitative perspective, an ability to maintain nitrogen balance at different levels of protein intake has been critical to this understanding. Furthermore, dietary protein and amino acid digestibility, the requirements of the indispensable amino acids, the Food and Agricultural Organisation/World Health Organisation/United Nations University (FAO/WHO/UNU) reference pattern of amino acids and identification of the limiting amino acid(s) has formed the foundation of human protein nutrition [55,56]. Based on this extensive research, current dietary references values (DRVs) and recommended daily allowances (RDA) for protein (as well as for energy and other nutrients) have been established. Depending upon the source of data, the current adult reference nutrient intake (RNI, +2 S.D. above the estimated average requirement EAR) for protein is set between 0.75 and 0.8 g/kg body weight/day [55,57]. This figure is sufficient to meet the needs of 97.5% of the adult population. Despite habitual daily intakes tending to be above this level, there has been an ongoing debate whether this RNI may be insufficient for some population groups including the elderly [58]. As SMM loss occurs with ageing and some elderly are vulnerable to the risk of sarcopenia, a higher daily intake of protein may be required to minimise loss of muscle in this population group. A second population for whom this RNI may be insufficient to meet their needs are endurance and strength-based athletes, where the need to offset the catabolic effects of endurance exercise and to maintain or build muscle mass must be met through dietary protein [23]. As reviewed above, individuals undergoing weight management intervention through a dietary energy restriction approach are also vulnerable to loss of SM, which may be exacerbated in the elderly population, especially if they have lower levels of physical activity and who may not be able to incorporate types of exercise that could benefit the preservation of SMM. Whilst it has been demonstrated that this loss could be ameliorated, in part by including an exercise element to the weight management programme, a higher proportion of energy intake derived from protein could also contribute to a greater preservation of FFM and SM [45]. Additionally, the quality of this additional protein is likely to be of equal importance [59]. Finally, it may be that a combination of an exercise element with a higher daily intake of protein could further protect from loss of SMM. An evaluation of the literature in this area now follows, with recommendations for protein intake where the evidence is supporting.

The idea that higher intakes of protein (absolute or proportional) may be an optimum dietary weight management strategy has found support in the scientific literature over a number of years. However, the focus of attention has tended to be directed towards the ability of dietary protein to suppress hunger and promote satiety [60,61], its effect on energy intake, resting metabolic rate, and thermogenesis [62], and endocrine and gut hormone responses [63]. Alongside these important areas of weight management, the role of dietary protein in the preservation of FFM and SMM under conditions of negative energy balance induced by an energy restricted diet has also been explored in some detail. This has been studied across a range of population groups including older people, those with components of metabolic disease, and sports people. In addition, certain studies have focused on the effect of specific protein-rich foods and meal replacement products as well as the timing of dietary protein provision. In addition, most of these studies have tended to include an exercise element, and so any body composition outcomes need to be interpreted in this context and where possible, the interaction and separate impacts of dietary protein and exercise will be clarified. Furthermore, variation in sample size, study design, and duration can make the comparison and interpretation of findings more difficult [34,64].

While most attention has been directed towards the effects of higher intakes of dietary protein, a study by Bopp et al. (2008) evaluated protein intakes ranging from below the RNI/RDA (0.47 g/kg body weight/day) up to the RDA of 0.8 g/kg body weight/day, with an energy deficit of approximately 2800 kcal/week [64]. Participants were post-menopausal women aged 50–70 y and the intervention lasted for 20 weeks. Body composition was assessed using DXA, which showed that loss of lean mass and appendicular lean mass was greater for those with the lower intake of protein. The authors concluded that inadequate protein intake during caloric restriction may be associated with adverse body-composition changes in postmenopausal women. As protein intakes did not exceed the RDA of 0.8 g/kg body weight/day, any potential beneficial effects of higher protein intakes on the preservation of FFM/SMM could not be inferred. Nevertheless, several studies have explored the effects of protein intakes greater than the RDA in a weight management programme for participants with excess weight. Earlier studies have tended to focus on FFM rather than SMM, which has been a feature of more research studies. One such study [34] that evaluated a high protein (~1.4 g/kg body weight/day) intake as part of an energy-restricted weight loss diet in women for 12 weeks, which did not include an exercise element, demonstrated a greater preservation of the lean body mass (assessed by DXA) compared with those fed at ~0.8 g/kg body weight/day, at similar levels of total body weight loss and it was suggested that obese individuals may be more susceptible to the negative effects of consuming a ‘normal’ level of protein during an energy-restricted weight management programme. A 2013 randomised controlled study evaluated the effect of dietary protein fed at three levels—0.8, 1.6, and 2.4 g/kg body weight/day—for 31 days in a group of 39 physically active military personnel (mean age 21 y) with a BMI between 22 and 29 kg/m^2^ [65]. After 10 days of a weight maintenance phase, 21 days of an energy deficit state were elicited by a restriction of energy intake by 30% together with an increase in physical activity of 10% above total daily energy expenditure. Body composition was assessed using DXA. With a mean loss of body weight of 3.2 ± 0.2 kg, the proportion of weight loss from the FFM was lower in those fed at 1.6 g and 2.4 g protein/kg body weight/day compared with the control group fed at the RDA. The highest level of protein (3xRDA) fed was no better than the 2xRDA at protecting FFM loss. In both these groups, loss of FM was greater than for the control group. While SMM was not directly measured, anabolic sensitivity of SM was assessed and was shown to be greater in those fed the higher levels of protein. In interpreting these findings, it is important to note that the participants were relatively young, physically active, military personnel, some with a BMI within the healthy range and exposed to an acute weight reduction period. How these findings translate to the general and obese populations is unclear. A study performed on a similar age group of men but with a mean BMI of 29.6 and for a similar duration was undertaken by Longland et al. in 2016 [66]. Two levels of protein (1.2 kg/kg body weight/d versus 2.4 g/kg body weight/d were fed as part of a hypocaloric (~40% reduction compared with requirements) diet. Resistance exercise and high-intensity interval training formed part of the protocol and body composition was assessed using a 4-compartment model. A significantly greater gain in LBM (*p* < 0.05) was observed in the group fed the higher level of protein coupled with a significantly greater loss in FM (*p* < 0.05). However, the study was unable to identify whether the gain in LBM was due to gains in SM. Finally, a 2020 study evaluated a low-energy, moderate carbohydrate (MCD) dietary approach on weight loss, blood lipids, and body composition over a 4-week period in a group of middle aged overweight and obese men [67]. In the diet prescription, protein was fed at 1.42 g/kg body weight/day (compared with 0.96 g/kg body weight/day in the group fed a low-energy mixed diet. BIA was used to quantify body composition, which showed a greater loss of body fat and a greater preservation of SMM compared with those fed the low-energy, mixed diet. Taken together, these findings suggest a role for a higher intake of dietary protein in preserving FFM/SMM during weight loss.

## 8. Metabolic Disease

Given the importance of SM as a marker for metabolic health/risk, ensuring the preservation of this tissue in individuals with excess weight and undergoing weight management is paramount. This has been evaluated in a select number of studies. In patients with type 2 diabetes, similar findings on FFM assessed using DXA have been found [68]. Here, a resistance form of exercise was included in the 16-week intervention where protein was fed as part of an energy restricted diet at a level of ~1.12 g/kg body weight/day. Overall weight loss was much greater in the high-protein-resistance training group, indicating a preferential loss of FM and a greater retention of FFM for the degree of overall weight loss. Furthermore, muscle strength remained superior in the high-protein-resistance training group compared with the other dietary and exercise groups, suggesting a potentially better metabolic fitness in these patients with type 2 diabetes. However, it should be noted that the body composition measurements formed only a small aspect of the study, which evaluated a whole range of glycaemic control and CVD risk markers. Concern was raised about the potential adverse effects of high protein diets on renal function, particularly in a diabetic population, although they suggested that a hypocaloric high-protein diet does not adversely affect renal function, at least in the short term, in individuals without overt renal impairment Furthermore, a 2019 systematic review suggested that high-protein diets potentially have beneficial effects for glycaemic control in T2DM without a detrimental effect on renal function or cardiovascular outcomes. Of the studies reviewed, there was no consistent evidence of benefit or of harm for kidney function [69]. In patients with chronic kidney disease, it has been recommended that high-protein diets are probably best avoided [70]. When evaluating the effect of higher intake of dietary protein on body composition in patients with metabolic syndrome (MetS), weight loss may not necessarily comprise part of the study. Campbell et al. (2015) evaluated a group of overweight and obese middle-aged adults with MetS undertaking an exercise (resistance and aerobic) training programme for 36 weeks while consuming an unrestricted diet together with a daily supplement of 200 kcal containing whey protein at 0, 10, 20, or 30 g [71]. Air-displacement plethysmography was used to determine body composition. Protein intake at >1.0 to <1.2 g/kg body weight/day (combined with resistance/aerobic training) resulted in a greater decrease in FM and an increased percentage of FFM compared with the lowest intake of protein, although the gain in FFM was not significantly different between groups. Furthermore, MetS indices were not influenced by the higher protein intakes. In the context of insulin sensitivity following weight loss and high intake of dietary protein, the positive effects on the preservation of FFM is not necessarily reflected in changes in metabolic function. Smith et al. (2016) compared the effects of weight loss of approximately 10% following a hypocaloric diet containing either 0.8 g or 1.2 g/kg body weight/day on body composition and muscle insulin action in obese post-menopausal women [72]. Again, a preservation of FFM was observed at the higher intake of protein (decline reduced by ~45% compared with the lower intake of protein. However, the higher intake of protein prevented the weight loss-induced improvements in muscle insulin signalling and insulin-stimulated glucose uptake. Such findings would have implications for the consideration of dietary macronutrient composition in a dietary weight management programme. 

## 9. Older Individuals and Post-Menopausal Women

Given the risk of SM loss in older age and its implications for sarcopenia, quality of life and independent living, many studies evaluating a higher protein intake on body composition during weight management have been undertaken in this age group. Backx et al. (2016) compared an intake of protein at 0.9 g and 1.7 g/kg body weight/day during a 12-week weight management intervention with a 25% restriction of energy intake in a group of overweight and obese participants with a mean age of 63 ± 5 years [73]. No exercise component was included in the intervention. Body composition was assessed by DXA and a leg muscle strength test was performed. Weight loss was similar between groups (~−9.0 kg) and there was a tendency for LBM to decline less in the group fed the higher protein level, although this did not reach statistical significance. Leg strength declined to a similar extent on both treatment groups. On this occasion, increasing protein intake above habitual levels did not preserve LBM. Similar findings have been observed by Verreijen et al. (2017) in older individuals following a hypocaloric weight loss programme for 10 weeks [74]. A 2 × 2 factorial design study evaluated protein fed at 2 levels: 0.8 and 1.3 g/kg body weight/day with or without a resistance exercise element. Body composition was assessed by air-displacement plethysmography. Neither the high protein intake nor the resistance exercise significantly affected body weight or composition changes compared with the control group, however, a within group analysis showed that the combination of a high protein intake with exercise caused a small but significant increase in FFM. It should be noted that the higher intake of protein only achieved a level of 1.13 g/kg body weight/day through normal food products. This has been suggested as a possible reason why no effect on the preservation of SMM was observed. These findings contrast with those of Beavers et al. (2017) and those of Sammarco et al. (2017) [75,76]. In the former study, using DXA as the body composition assessment instrument, a higher protein intake of 1.2–1.5 g/kg body weight/d was evaluated. This formed an element of a weight management programme incorporating a meal replacement approach within a healthy eating plan providing 1100–1300 kcal/d and 120–150 g protein. Participants had a mean age of mean age 70.3 y. These were compared against a weight stable group over a period of six months. Total weight loss was greater in the intervention group. However, despite a small decline in FFM, a differential treatment effect was not observed for this body compartment. Thus, using a high-protein diet as part of a meal replacement approach produced clinically meaningful weight and fat loss while helping to preserve lean tissue mass. The latter study was based on a small sample size and body composition was assessed by bioimpedance analysis. However, with a hypocaloric, high protein (1.2–1.4 g/kg body weight/d) diet followed for four months, a greater loss of FM and a gain in FFM was observed. On the balance of evidence, there appears to be a beneficial effect for the preservation of FFM in older individuals by consuming a higher intake of protein when following a weight management programme. The dietary impact appears to augment the effect of resistance exercise with or without an aerobic exercise element. Most of these studies, however, have tended to assess FFM/LBM rather than SMM and it is SM preservation that is key to metabolic health and physical function [2,3,4]. 

## 10. Protein-Rich Foods and Preservation of FFM

Thus far, the reviewed studies have not tended to focus upon specific protein-rich foods but rather on an overall higher intake of dietary protein above the RDA. However, the protein quality of the diet should not be overlooked since the presence and pattern of the essential amino acids in the diet are crucial when considering the impact of dietary protein on skeletal muscle synthesis and preservation of SMM during a hypocaloric weight management programme [56,59,62,77]. In this context, the contribution of lean meat and fish, because of their amino acid profiles and protein quality scores, must be recognised [59,77]. Additionally, the role of specific protein-rich foods with a high quality have been evaluated, especially whey protein [78,79,80]. Whey is a key milk protein rich in essential amino acids, particularly leucine, and is a popular supplement used in sports and exercise nutrition. Despite its high quality, conflicting findings have been observed when whey protein has been used and studies have been compromised, in part, by small sample sizes [79]. One such study conducted in 2012 (Coker et al., 2012) found only a modest preservation of lean tissue in an eight-week hypocaloric weight loss study on elderly participants fed a supplement of whey protein plus essential amino acids, compared with a control group fed a competitive meal replacement product [78]. Equally, albeit on a small scale study, a daily whey protein supplement added to a very low-calorie diet for four weeks did not lead to any significant difference in pre- and post-FFM measurements (assessed by DXA) in a group of obese participants undergoing a weight management intervention [79]. However, a greater loss of FM was observed with the whey protein formula. Similarly, a study by Smith et al. (2018) evaluated a leucine-rich whey protein formula (1.2 g protein/kg body weight/d versus a weight maintenance and weight loss group fed at 0.8 g/kg body weight/d) in a weight loss intervention in middle-aged post-menopausal women [80]. They failed to demonstrate any clinically important therapeutic effects on muscle mass (assessed by DXA) or strength, despite a small but significant decline in lean mass following moderate weight loss. In contrast, a high whey protein–, leucine-, and vitamin D–enriched supplement (1.11 g protein/kg body weight/d) compared with an isoenergetic control (0.85 g protein/kg body weight/d) preserved and enhanced appendicular muscle mass (assessed by DXA) in obese older adults during a hypocaloric diet and resistance exercise programme for 13 weeks [81]. This is one of only a small number of studies that have specifically quantified appendicular muscle mass. However, muscle strength and physical performance were improved in both the intervention and control groups. Their findings support the suggestion that intake of high-quality protein should be increased during a weight loss intervention in obese older adults to assist in the prevention of weight loss-induced sarcopenia.

Other protein-rich foods that have been evaluated include dairy products and whole eggs [82,83]. Josse et al. (2011) evaluated the impact of three levels of dairy consumption in combination with high or adequate intakes of protein [82]. These were combined with a modest energy intake reduction and an exercise element (aerobic and resistance) to assess weight loss and body composition (measured by DXA) in healthy premenopausal overweight and obese women over a period of 16 weeks. While all groups lost weight, the high protein-high dairy group gained more lean tissue compared with the adequate protein-medium dairy, which maintained lean mass. The adequate protein-low dairy group lost lean mass. These findings suggest that a higher protein and increased dairy product intake promotes a more favourable body composition as well as strength and fitness. The relevant beneficial nutritional profile of the low-fat dairy foods is likely high-quality protein together with adequate calcium. The inclusion of three whole eggs (high-protein, 1.4 g/kg body weight/d) in the diet has been evaluated in a 12-week weight loss intervention compared with a normal protein (0.8 g/kg body weight/d) in a group of older individuals [83]. While total body weight was reduced in both groups, and lean mass (assessed by DXA) was preserved in the high-protein diet, suggesting that a high-protein diet based around the consumption of whole eggs is a viable whole food approach to increase total protein intake and to promote lean mass retention during a weight management intervention in older people. It should be noted that the sample size was small and so caution must be exercised when interpreting the findings. However, it is worth reiterating the high quality of the protein present in hens’ eggs and is likely to be important for essential amino acid provision and retention of lean mass in this population group. Equally, it should be recognised that eggs and milk can be allergenic in susceptible individuals and must be considered if these foods are used in this dietary approach to weight loss and maintenance of SMM [84].

Extreme energy restricted diets include the very low-calorie diet (VLCD), which, as a total meal replacement and when used under medical supervision, can result in rapid weight loss. Such a dietary approach is being rigorously evaluated in primary care settings including in patients with T2DM, with some evidence of remission of the condition following such an extreme dietary intervention [85,86]. However, because of the rate of weight loss, tissue loss can equally be derived from both FM and FFM, and likely SM. Thus, increasing the proportion of energy from protein in this type of diet could result in better preservation of SMM and a more preferential loss of FM. One study compared a VLCD ketogenic diet (450–500 kcal/d for women, 650–700 kcal/d men, 55–60% energy from protein) supplemented with amino acids with a VLCD alone on weight loss and muscle mass (assessed by DXA) [87]. In a small group (*n* = 25) of adults with a mean age of 47.5 y, after three weeks, weight loss was greater in the VLCD group and whereas total body lean mass decreased in this group, in the amino acid supplemented group, no significant difference in total body lean from the baseline was observed. It should be noted this was a small-scale study and caution should be exercised when interpreting these findings. However, further research on VLCD and meal replacement products is warranted, especially as an increasing number of meal replacement products are available and some have been evaluated in depth in weight loss studies focusing upon body composition, metabolic, and inflammatory measures [88,89,90,91,92].

## 11. Timing of Protein Feeding and Muscle Mass Maintenance

An area of protein nutrition and weight loss that has been under-researched is that of the timing of meals and the spacing out of dietary protein provision across the day. Protein pacing is the term used to describe the spacing out of intake across six meals/d. This approach was used with a hypocaloric (~25% energy deficit) in obese individuals over a 12-week (weight loss) and 52-week (weight maintenance) intervention period and compared with a three meal/d approach [93]. Over the short term (12 weeks), those following the protein-pacing diet (0.3 g protein/kg body weight per meal, >1.4 g protein/kg body weight/d) demonstrated similar changes in body weight and composition (lean body mass). However, over the longer term, the protein pacing group were more efficacious at improving body weight and composition (LBM). More research is required to clarify any short- or long-term benefit for preserving SMM using this protein pacing approach.

## 12. Weight Loss in Sports People

A key population group for whom weight loss at certain time points may be important are those engaged in sport at professional or serious levels. This group includes those involved in combat sports such as boxing, jockeys, and sports such as rowing. At certain timepoints, these athletes could engage in rapid weight loss strategies that could potentially be harmful to their health. Nevertheless, weight loss may be a requirement for qualification to compete in a lower weight group category or for riding a racehorse that has been allocated a lighter weight to carry. In situations such as these, it is especially important to provide an adequate supply of protein to them for the maintenance of SMM, as a key risk factor for these individuals is the potential loss of lean tissue, which is significant in maintaining performance in both resistance and aerobic forms of exercise. It can be a challenge to consume a diet to meet the energy and carbohydrate requirements for training and competition whilst attempting to lose body fat and maintain SMM. Hence a small number of studies have evaluated the role of higher intakes of protein coupled with a hypocaloric diet in competitive sports people. Despite the potential attraction of rapid weight loss strategies, the literature suggests that a slower rate of weight loss is preferable in this population group as this approach tends to prioritise fat loss over loss of lean tissue [94,95,96]. In addition, there is some suggestion that protein intakes significantly beyond current recommendations (1.6–2.4 g protein/kg body weight/d) [97] for athletic populations may result in improved body composition [95]. As evidence for this, Mettler et al. (2010) evaluated the effect of increased protein intakes on LBM loss (assessed by DXA) during short-term hypocaloric weight loss in athletes [98]. They observed that a protein intake of ~2.3 g/kg body weight/d (35% of energy intake) was significantly superior to ~1.0 g/kg body weight/d (~15% energy intake) in order to maintain LBM during short-term hypocaloric weight loss. This intake is indeed at a completely different level and considered extreme compared with weight loss studies conducted in the general population. Any potential negative consequences of such high protein intakes should be considered in future studies. Similar to the protein pacing approach, timed ingestion of protein, as close as possible following exercise, coupled with a higher overall daily dietary protein intake could be one strategy to minimise loss of SMM when undergoing a weight loss programme. A window of opportunity exists immediately post exercise (recovery phase) whereby consuming protein-rich food at that time can capitalise upon the maximal rate of muscle protein synthesis (MPS). Again, a relative hyperleucinaemia at this time appears to drive this MPS [25]. However, more research is needed to evaluate this suggestion but could be of benefit to some athletes. Finally, there is some preliminary evidence to suggest that a low-carbohydrate diet may be beneficial for athletes who want to lose body weight and fat, without compromising muscle mass [99].

An often-overlooked group of athletes with body weight issues are those who have reached the end of their sporting career, especially those who have developed muscularity and strength for performance. A reduction in post-career physical activity as well as a failure to modify dietary intake can lead to excess weight gain, especially in the FM compartment. This could eventually lead to developing components of metabolic disease and increasing health risk. A small number of studies have begun to explore optimal weight loss strategies for health in former athletes. Dietary approaches have included various degrees of energy intake reduction and a Mediterranean-type diet. Outcomes with respect to weight reduction and FFM/SMM preservation have been shown to be promising in these preliminary studies [100,101].

## 13. Conclusions

In this review, the role of dietary protein and exercise as a means for minimising loss of lean tissue, particularly SM during weight management interventions across a range of population groups, was evaluated. Arriving at a definitive conclusion can be difficult for a number of reasons and the routine issues of study design, sample size, duration of intervention, population group, and assessment instruments all impact upon the findings. However, on balance, there appears to be sufficient evidence to suggest that a greater contribution of dietary protein, above the current RNI/RDA, may be beneficial in preserving SMM when undergoing a weight management intervention. This may be particularly important for older individuals who have the added risk of age-related loss of SMM and strength (sarcopenia), who may also need to lose body weight and body fat. It is interesting to note that a recent analysis of dietary protein intake in the UK elderly population (aged 65–89 y) demonstrated that fewer than 50% of the participants met the current UK RNI of 0.75 g/kg body weight/d [102]. This links with the suggestion that an optimal intake of dietary protein could be around 1.2 g/kg body weight/d [58] as well as the ESPEN endorsed recommendation of at least 1.0–1.2 g protein/kg body weight/day for healthy older people [103]. In the meantime, current clinical guidelines in the UK [1] do not address the loss of FFM whilst following a weight loss programme and continue to advocate consuming less dietary energy than energy expenditure, achieved through a low-fat, 600 kcal/d deficit. No reference is made to proportionately higher intakes of protein, but it may now be time to revisit current weight management guidelines in light of the evidence on dietary protein and preservation of SMM. In support of these dietary protein suggestions, findings from a recent systematic review and meta-analysis of randomised controlled studies concluded that higher intakes of dietary protein were associated with better cardiometabolic risk factors including systolic blood pressure, some lipid outcomes, and insulin levels as well as weight loss and fat loss [104]. However, consideration must be made as to how, in practical terms, a higher intake of protein can be achieved when concurrently reducing energy intake. One possibility is a supplement approach, similar in composition to meal replacement products, as some of these can be naturally high protein, low fat, low energy products as well as being rich in micronutrients. This approach could assist with the ‘protein pacing’ strategy, which is also a recommendation to help improve meal-induced stimulation of MPS in the elderly [103]. The protein quality of the supplement should also be considered. As noted earlier in this review, the essential amino acid leucine is key to optimal stimulation of MPS post-prandially. This is also true for the elderly [103]. The relative merits of plant versus animal sources of protein should also be considered. Longer term care should be exercised if following this dietary approach, as individuals should ensure sufficient daily intake of fruits, vegetables, and fibre-rich foods, given their low energy density but nutrient rich characteristics.

## Data Availability

Not applicable.
